# Inspiratory and peripheral muscle strength as predictors for extubation failure in COVID-19 patients

**DOI:** 10.1186/s13054-022-03926-0

**Published:** 2022-02-15

**Authors:** Shraddha Shah

**Affiliations:** Department of Physiotherapy, Manipal College of Health Professions, Manipal Academy of Higher Education, Manipal, Karnataka 576104 India

## Dear editor,

With great interest, I read the article by Fleuren et al. on determining the predictors for extubation failure in COVID-19 patients [1]. The authors have methodically utilised the machine learning models to identify the potential parameters that would predict the extubation failure in critically ill COVID-19 patients. They discuss the potential predictors selected by a team of intensivists for modeling from the Dutch Data Warehouse (DDW). With this study, they concluded that the individual ventilatory characteristics, inflammatory markers, neurological status and body mass index (BMI) were the most important parameters to predict extubation failure in COVID-19 patients.

However, as the article discusses the indicators for predicting extubation failure, it becomes essential to consider the inspiratory muscle strength and gross peripheral muscle strength as independent indicators for predicting extubation. Extubation is a complicated process and requires a balance between global respiratory load and ability to overcome this load for separation from invasive mechanical ventilation (IMV). Evidence from the meta-analysis carried out shows that non-COVID-19 patients receiving IMV in ICU have a high risk of developing respiratory and limb muscle weakness [2]. Similarly, considering the novelty of the disease, there is sufficient literature available stating that patients with severe COVID-19 requiring prolonged ICU stay and IMV have been diagnosed with ICU-acquired weakness (ICUAW) [3]. Further, from the growing evidence, it has been observed that poor peripheral muscle strength, particularly quadriceps muscle, is effective indicator (*p* = 0.02) in predicting extubation failure in critically ill patients [4]. We have also observed that in non-COVID-19 patients, inspiratory muscle training (IMT) achieved either by threshold pressure training or adjusting ventilator sensitivity, did not directly predict extubation outcome; however, it concluded that IMT significantly improved maximal inspiratory pressure (MIP) (MD 7 cmH_2_O, 95% CI 5–9), rapid shallow breathing index (RSBI) (MD 15 breaths/minute/l, 95% CI 8–23) and weaning success (RR 1.34, 95% CI 1.02–1.76) [5].

In closing, I believe that inspiratory and peripheral muscle testing can be added to the variables listed by Fleuren et al. to predict extubation failure and avoid the serious sequelae caused by re-intubation in COVID-19 patients. Once again, I commend the authors for their excellent work in this area, which remains to be the need of the hour.

## Data Availability

Not applicable.
